# Enhancing Omega-3 Long-Chain Polyunsaturated Fatty Acid Content of Dairy-Derived Foods for Human Consumption

**DOI:** 10.3390/nu11040743

**Published:** 2019-03-29

**Authors:** Quang V. Nguyen, Bunmi S. Malau-Aduli, John Cavalieri, Peter D. Nichols, Aduli E. O. Malau-Aduli

**Affiliations:** 1 Animal Genetics and Nutrition, Veterinary Sciences Discipline, College of Public Health, Medical and Veterinary Sciences, Division of Tropical Health and Medicine, James Cook University, Townsville, QLD 4811, Australia; quang.nguyen2@my.jcu.edu.au (Q.V.N.); john.cavalieri@jcu.edu.au (J.C.); Peter.Nichols@csiro.au (P.D.N.); 2 College of Economics and Techniques, Thai Nguyen University, Thai Nguyen 252166, Vietnam; 3 College of Medicine and Dentistry, Division of Tropical Health and Medicine, James Cook University, Townsville, QLD 4811, Australia; bunmi.malauaduli@jcu.edu.au; 4 CSIRO Oceans & Atmosphere, P.O. Box 1538, Hobart, TAS 7001, Australia; 5 Nutrition Society of Australia (NSA), Level 3, 33-35 Atchison Street, St Leonards, NSW 2065, Australia; 6 Australasian Section, American Oil Chemists Society (AAOCS), 2710 S. Boulder, Urbana, IL 61802-6996, USA; 7 Asia Pacific Nutrigenomics and Nutrigenetics Organisation (APNNO), CSIRO Food & Nutrition, Adelaide, SA 5000, Australia

**Keywords:** dairy-derived foods, n-3 LC-PUFA, milk, cheese, lipids, oil, nutritional supplementation, genetic manipulation, candidate genes, FADS

## Abstract

Omega-3 polyunsaturated fatty acids (n-3 PUFA) are termed essential fatty acids because they cannot be synthesized de novo by humans due to the lack of delta-12 and delta-15 desaturase enzymes and must therefore be acquired from the diet. n-3 PUFA include α-linolenic acid (ALA, 18:3n-3), eicosapentaenoic (EPA, 20:5n-3), docosahexaenoic (DHA, 22:6n-3), and the less recognized docosapentaenoic acid (DPA, 22:5n-3). The three long-chain (≥C_20_) n-3 PUFA (n-3 LC-PUFA), EPA, DHA, and DPA play an important role in human health by reducing the risk of chronic diseases. Up to the present time, seafood, and in particular, fish oil-derived products, have been the richest sources of n-3 LC-PUFA. The human diet generally contains insufficient amounts of these essential FA due largely to the low consumption of seafood. This issue provides opportunities to enrich the content of n-3 PUFA in other common food groups. Milk and milk products have traditionally been a major component of human diets, but are also among some of the poorest sources of n-3 PUFA. Consideration of the high consumption of milk and its processed products worldwide and the human health benefits has led to a large number of studies targeting the enhancement of n-3 PUFA content in dairy products. The main objective of this review was to evaluate the major strategies that have been employed to enhance n-3 PUFA content in dairy products and to unravel potential knowledge gaps for further research on this topic. Nutritional manipulation to date has been the main approach for altering milk fatty acids (FA) in ruminants. However, the main challenge is ruminal biohydrogenation in which dietary PUFA are hydrogenated into monounsaturated FA and/or ultimately, saturated FA, due to rumen microbial activities. The inclusion of oil seed and vegetable oil in dairy animal diets significantly elevates ALA content, while the addition of rumen-protected marine-derived supplements is the most effective way to increase the concentration of EPA, DHA, and DPA in dairy products. In our view, the mechanisms of n-3 LC-PUFA biosynthesis pathway from ALA and the biohydrogenation of individual n-3 LC-PUFA in ruminants need to be better elucidated. Identified knowledge gaps regarding the activities of candidate genes regulating the concentrations of n-3 PUFA and the responses of ruminants to specific lipid supplementation regimes are also critical to a greater understanding of nutrition-genetics interactions driving lipid metabolism.

## 1. Introduction

Omega-3 polyunsaturated fatty acids (n-3 PUFA) contain more than two double bonds with the first double bond on the third carbon atom from the methyl end of the molecule. The common types of n-3 PUFA are: Shorter chain (SC, ≤C_18_) n-3 PUFA including α-linolenic acid (ALA, 18:3n-3) and stearidonic acid (SDA, 18:4n-3), and long-chain (≥C_20_) n-3 PUFA (n-3 LC-PUFA) including eicosapentaenoic (EPA, 20:5n-3); docosahexaenoic (DHA, 22:6n-3); and the less studied docosapentaenoic (DPA, 22:5n-3) acids [1]. The focus herein is on LC-PUFA due to their beneficial effects in human pathologies. Since Bang et al. [2] first demonstrated the positive relationship between low amounts of some non-communicable diseases and high n-3 LC-PUFA consumption of the Eskimos, numerous studies have consistently demonstrated their vital role in inhibiting major chronic diseases [3], including adipogenic, diabetogenic, atherogenic [4], inflammatory [5,6] and carcinogenic [7,8] diseases. Moreover, high consumption of n-3 LC-PUFA is typically associated with a higher cognitive performance and a lower risk of developing Alzheimer’s disease [9,10,11]. Previous studies on n-3 LC-PUFA have focused mainly on EPA and DHA, but not DPA despite its structural and beneficial effects on human health being similar to those of EPA and DHA [12]. The unavailability of pure DPA as a commercial product for performing clinical and nutritional trials is one possible explanation for this shortcoming. The term n-3 LC-PUFA in this current review includes EPA, DHA, and DPA.

Chronic or non-communicable diseases have remained as the most leading cause of death worldwide, with 41 million deaths accounting for 71% of reported global deaths (57 million) [13]. This report also indicated that an unhealthy diet with low intake of n-3 LC-PUFA, continues to be one of the main factors that either directly or indirectly induce chronic diseases. Although there is a general awareness that fish and seafood are the dominant source of n-3 LC-PUFA, seafood consumption is still insufficient, thus the human diet persists with low n-3 PUFA intake [14]. The traditional diet often does not contain regular consumption of fish and marine products, especially in Western countries [12,15]. When taken together with the often high cost of seafood [16], these combined factors probably have been the major grounds for this trend. In contrast, milk and its processed products are known as poor sources of n-3 LC-PUFA content [17], although they have played an important role in human diets for more than 8000 years [18]. This is because dairy foods are important sources of energy, protein, fat, and vital microelements including calcium, vitamin D and potassium for humans [19,20]. According to the OECD/FAO report [21], the 2015 global consumption of milk and dairy products was 111.3 kg per capita, and is expected to increase by approximately 12.5% by 2025. This fact has led to a number of studies focusing on enhancing the beneficial n-3 PUFA and n-3 LC-PUFA in milk and its processed products, mostly from cows and sheep, for human consumption [17]. The aim of the present review was, therefore, to evaluate and update the published literature on the effects of n-3 LC-PUFA on human health and to also examine recent research on improving the concentrations of these health beneficial FA in dairy-derived foods. Consequently, outcomes from this review may open up opportunities for future further research into nutrition-genetics interactions influencing lipid metabolism in dairy-derived foods.

## 2. Metabolic Pathways, Human Health Benefits and Recommended Intake of n-3 PUFA

### 2.1. Dietary n-3 PUFA Intake Recommendations

Dietary intake recommendations of n-3 LC-PUFA from different organizations vary largely and also depend on many factors including age, gender, and consumption purposes of consumers [1,22]. Adhering to National Health and Medical Research Council (NHMRC) recommendations [23], the daily intakes of ALA and total EPA+DPA+DHA considered adequate for men are 1.3 g/day, and 160 mg/day, and for women, 0.8 g/day and 90 mg/day, respectively. These dietary requirements of n-3 PUFA are not optimal, but are seen as sufficient to prevent deficiency symptoms for adults. However, with the aim at reducing chronic disease risk, the NHMRC suggested that dietary intakes of total n-3 LC-PUFA of 430 mg/day for women, and 610 mg/day for men should be adequate to meet requirement levels. In order to prevent the risk of coronary heart disease, FAO and WHO [24] recommended sufficient daily intake of EPA + DHA at 250 mg for adult males and non-pregnant or/and non-lactating adult females, and at 300 mg for lactating and pregnant women. In the case of disease treatment, such as for hypertriglyceridemia patients who have high triglyceride level symptoms, a much higher intake of total EPA + DHA from 2–4 g/day is recommended by the American Heart Association [25]. A recent review by Nguyen et al. [22] stated that the intake recommendation of n-3 LC-PUFA for primary prevention of cardiovascular disease across all organizations is about 500 mg/day, which is equivalent to two or three servings of fish per week.

### 2.2. Metabolic Pathways for the Biosynthesis and Dietary Sources of n-3 PUFA

Due to the lack of delta-12 and delta-15 desaturase enzymes, mammals (including humans) cannot synthesize n-3 PUFA de novo, thus these essential FA must be acquired via foods or nutritional supplements [26]. The first step in the n-3 LC-PUFA synthesis pathway for the human body is the conversion of ALA to SDA, with ALA mostly acquired from green plant tissues and plant-derived oils, especially flaxseed/linseed and canola oil [27] (Table 1).

**Figure 1:**
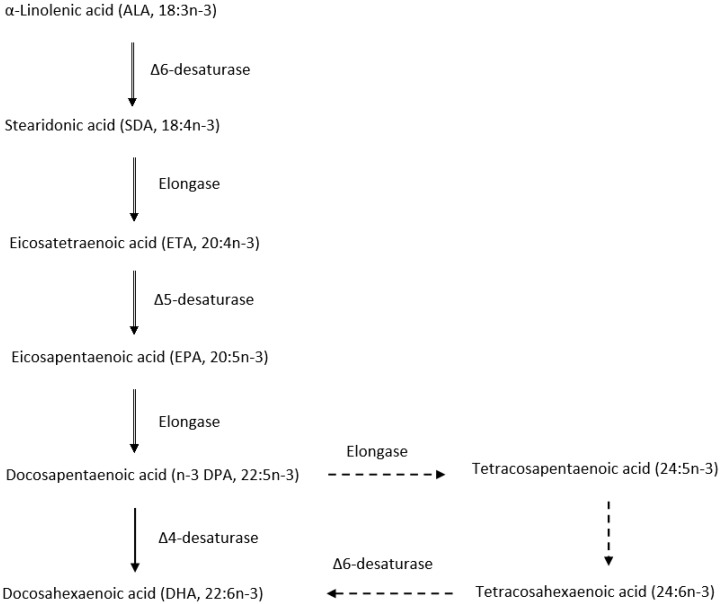
Possible biosynthesis and metabolic pathway of n-3 LC-PUFA. Thick arrows represent the conventional pathway; dotted lines with arrows represent presently accepted pathway (adapted from Park et al. [30] and Sprecher [29]).

There are two recognised biosynthesis pathways for n-3 LC-PUFA (Figure 1), including the presently accepted pathway [29] and conventional metabolic pathway [30]. In the former pathway, DHA was produced from DPA via sequential desaturation and elongation combined with a final β-oxidation where tetracosapentaenoic acid (24:5n-3) is chain-shortened by two carbons. The latter conventional metabolic pathway, in contrast, consists of direct conversion of DHA from DPA under the catalysis of delta-4 desaturase enzyme. The molecular evidence for delta-4 desaturase that supported the conventional metabolic pathway for n-3 LC-PUFA biosynthesis was first demonstrated by Park et al. [31]. Further research is needed to clarify the specific pathway for n-3 LC-PUFA biosynthesis in the human body, but most studies have confirmed a very low rate of conversion of ALA to n-3 LC-PUFA, in particular, to DHA (0.05% or less) [32]. The specific mechanism(s) by which biosynthesis of these essential FA occurs is limited in man and is still largely unknown. Calder [3] suggested that a possible cause for this limitation is the competition between biosynthetic pathways of ALA conversion to n-3 LC-PUFA and linoleic acid (18:2n-6) conversion to n-6 LC-PUFA as the two pathways employ the same set of enzymes. In addition, based on previous animal studies, deficiencies of insulin [33], protein [34] and microminerals [35] might lead to lower delta-6 desaturase enzyme activity, thus contributing to the low efficiency of this pathway.

Due to the limitation of n-3 LC-PUFA biosynthesis in the human body from ALA, the best way of acquiring these essential FA is from dietary sources [36]. Fish and seafood currently are the major sources of n-3 LC-PUFA with high concentration ranges across seafood species [1,37]. The average content of total n-3 LC-PUFA in 150 g wet weight of wild caught Australian fish, shellfish, prawns, and lobsters are 350, 250, 180, and 160 mg respectively, with a range of species also having markedly higher contents than these average values [1]. The level of these FA for the two common fish species farmed in Australia - Atlantic salmon, and barramundi - examined by Nichols et al. [38] are 980 and 790 mg/100 g, respectively. Compared to the previous results [1], the concentration of n-3 LC-PUFA for these farmed fish had decreased significantly by 50% or more. Changes in feed ingredients for farmed fish, in which fish meal and fish oils have been substituted by non-traditional oil sources such as plant and/or chicken oils were the reasons for this trend [38]. Foods derived from animals have much lower n-3 LC-PUFA content in comparison to marine products (Table 2).

### 2.3. n-3 LC-PUFA Consumption and Chronic Diseases

The biological functions of n-3 LC-PUFA are firstly represented by their occurrence in all cellular membranes in all tissues of the body, and in particular, at high content levels in the retina, brain, and myocardium [48,49]. For example, due to a high concentration of DHA in the membranes of the human retina and brain, it plays an important role in regulating membrane receptors, membrane-bound enzymes and transduction signals [48]. In addition, n-3 LC-PUFA have the potential to transform into a group of mediators such as the E-series and D-series resolvins at the expense of inflammation mediators from arachidonic acid (20:4n-6, ARA) which is the primary cause of various chronic disease treatments [49,50]. Chronic inflammation that persists for a long time has a strong link with the development of many chronic diseases including cancer, cardiovascular (CVD), neurodegenerative, and respiratory diseases [3,51]. Moreover, there is a positive correlation between n-3 PUFA dietary consumption and incorporation of these FA into cell membranes [52,53] that explains a positive effect of adequate dietary n-3 PUFA consumption on inhibiting chronic diseases.

Cardiovascular diseases refer to a collective term for heart and/or blood vessels related diseases that are by far, the most leading cause of mortality worldwide with 17.9 million deaths reported in 2018 [13]. Therefore, the effects of n-3 PUFA on major CVD including coronary heart disease (CHD) and stroke have been reported in numerous studies [54,55,56]. One of the potential roles of n-3 PUFA in reducing the risk of CHD is by counteracting many steps of atherosclerosis [57], the major cause of CHD [58]. Novel findings [59] demonstrated that enriched-DHA canola oil supplementation could reduce the risk of CHD by improving high-density lipoprotein cholesterol, triglycerides, and blood pressure. In addition, previous meta-analyses established the link between increasing intakes of n-3 LC-PUFA and reducing the risk of CHD death by 10–30% [54]. In terms of stroke, dietary consumption of n-3 PUFA can reduce the volume of ischemic stroke [60] by promoting antioxidant enzyme activities or partly acting as an antioxidant. n-3 PUFA can provide further benefits relating to stroke post-treatments [55], by generating other important responses such as neuranagenesis and revascularization. The latest meta-analysis of prospective cohort studies [61] supported a strong inverse relationship between daily fish intake and the risk of stroke. Following CVD, cancer is the second most common cause of death [13]. Clinical and epidemiological studies have demonstrated the role of n-3 LC-PUFA in either reducing the risk of developing cancer or improving chemotherapy outcomes in existing cancer patients of several common types of cancer [3,62]. Long-term studies by Kato et al. [63], Terry et al. [64] and Takezaki et al. [65] concluded that increased consumption of dietary n-3 LC-PUFA lowered the risk of colorectal, prostate and lung cancer, respectively. Van Blarigan et al. [66] also reported that higher intake of n-3 LC-PUFA improved disease-free survival by 28% in colon cancer patients. The effect of these PUFA is more varied. While Holmes et al. [56] showed no relation between fish consumption and breast cancer, recent studies confirmed the positive impact of n-3 fat on not only inhibiting [67,68], but also reducing fatigue [69], in breast cancer patients. In contrast to the large number of studies that confirmed the positive effects of n-3 PUFA on these two major chronic diseases, other research findings reported neutral, inconclusive or even possible negative effects [62]. For instance, there was no statistically significant association between major CVD events and n-3 PUFA supplementation based on a meta-analysis of previous randomized clinical trials [70]. Similarly, results from a large prospective cohort study by Rhee et al. [71] reported a neutral effect of n-3 PUFA intake on the risk of major CVD in healthy women aged ≥45 years. With respect to cancer, Holmes et al. [72] showed that there was no relationship between fish consumption and breast cancer, while in one case, the intake of n-3 PUFA was claimed to induce the risk of basal cell carcinoma on skin cancer [73].

Apart from CVD and cancer, large studies have recognised the role of n-3 LC-PUFA in regards to brain related cognitive treatments and other common chronic diseases such as rheumatoid arthritis, type-2 diabetes and obesity. Relating to brain issues in humans, bioactivities of n-3 LC-PUFA, particularly DHA, play an important role in neural membrane structure, neurotransmission, and signal transduction [74], and positive effects on treatment of different neurodegenerative and neurological disorders [75]. Lower n-3 PUFA intakes have been reported to induce the risk of Alzheimer’s disease [76], while increased fish oil intakes for Parkinson’s disease patients resulted in a significant reduction in depressive symptoms [77]. Examining rheumatoid arthritis, Abdulrazaq et al. [78] reported that a majority of studies confirmed the beneficial effect of utilising n-3 LC-PUFA at doses of 3-6 g/day on pain relief in patients. Findings on the benefits of n-3 PUFA consumption in type-2 diabetes and obesity remain inconsistent. While some authors have recognised that n-3 PUFA intake can reduce the incidence of diabetes [79,80], the findings from a systematic review and meta-analysis reported by Wu et al. [81] suggested a neutral effect of EPA + DHA and seafood consumption on the development of diabetes. Similarly, no significant relationship between n-3 PUFA and obesity was reported in the review by Albracht-Schulte et al. [82]. In contrast, high fish intake in men could lower the risk of being overweight [83], although an opposite result was observed in women with higher fish consumption [84].

The controversies regarding the role of n-3 PUFA in chronic diseases may be explained by many factors such as dose, duration, baseline intake [85], specific type of the chronic disease and risk group [86]. Due to this continuous debate and variations in experimental design, it has not been very evident from current scientific literature and medical opinion confirming or rejecting the beneficial effects of n-3 PUFA in reducing the risk of human chronic diseases [62]. Therefore, large and unified clinical trials need to be conducted to conclusively identify the exact role of n-3 PUFA as independent or supplementary factors in specific chronic diseases.

## 3. Lipid Metabolism in Ruminants: Obstacles to Enriching Milk Fat with n-3 PUFA

Since all of the long-chain FA in milk fat are derived from the absorption of fatty acids from the small intestine and body fat reserves that have both originated from dietary FA [17,87], manipulating the diet or feeding regime is the most popular way to alter milk fat composition. However, the efficiency of this approach in ruminants is still limited due to rumen microbial fermentation [88].

**Figure 2:**
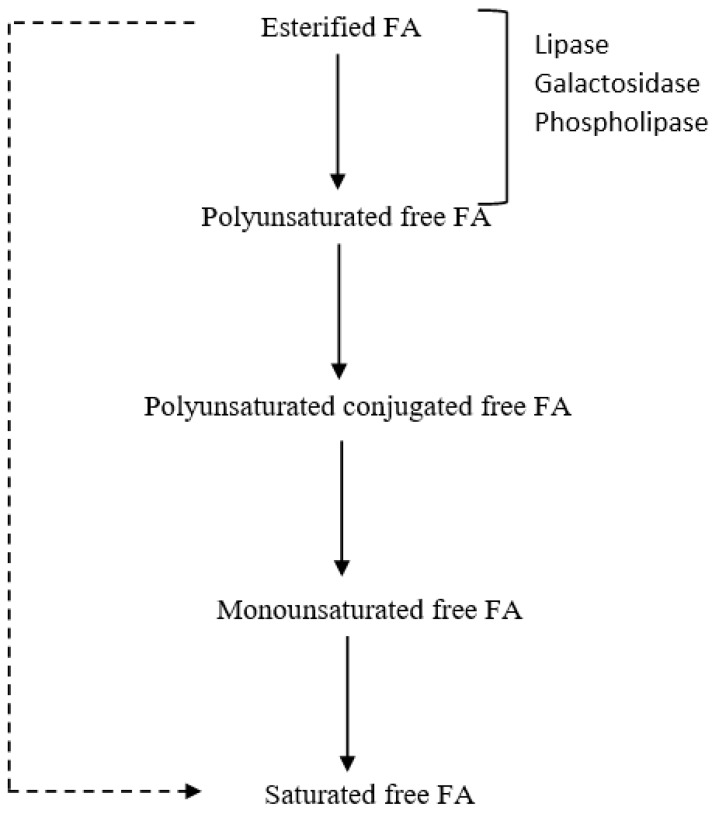
The scheme of lipolysis and biohydrogenation (adapted from Buccioni et al. [88]).

Dietary lipid sources for ruminants are mainly from forages, supplements or concentrates including cereal grains, oilseeds and animal fats. Lipids derived from forages contain largely glycolipids and phospholipids, while triglycerides are found primarily in supplements [89]. Once dietary lipids enter the rumen, lipolysis occurs and it involves hydrolysis of ester linkages to release free fatty acids for the next biohydrogenation (BH) process [88] (Figure 2).

**Figure 3:**
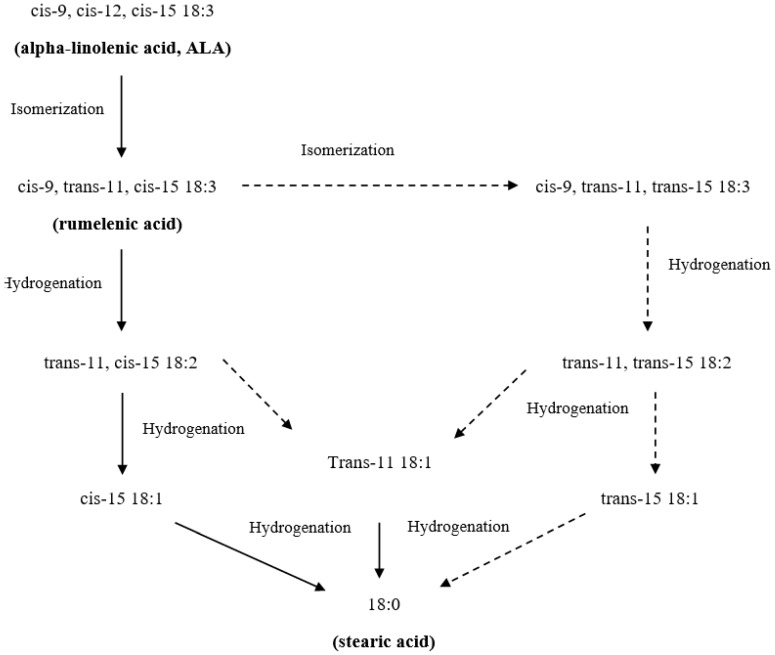
Ruminal biohydrogenation of alpha-linolenic acid. Thick arrows represent the major pathway; dotted lines with arrows represent putative pathway (adapted from Gomez-Cortes et al. [108]).

**Figure 4:**
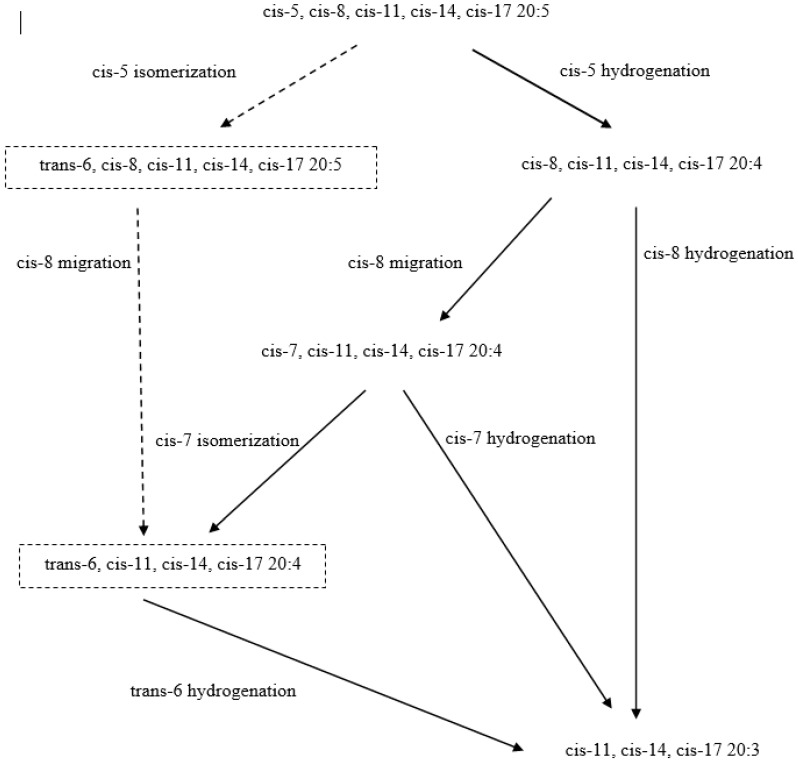
Possible biohydrogenation pathways of 20:5n-3. Solid arrows represent possible major pathway; dotted lines with arrows represents hypothetical pathway (adapted from Toral et al. [95]).

Under the activity of rumen microbes, unsaturated fatty acids (UFA) including PUFA are hydrogenated to monounsaturated FA (MUFA) and ultimately, saturated FA (SFA) through the addition of a double bond of two hydrogen atoms. The principal role of this process is to maintain a stable rumen environment by reducing the toxic effects of free UFA on bacterial growth in the rumen [89]. Due to the high rate of hydrolysis and BH, only small amounts of PUFA from the diet can pass through the rumen into the duodenum for absorption [90]. According to Shingfield et al. [91], dietary ALA in the rumen can be hydrogenated into 18:0 (Figure 3) at the rate of 85% to 100%. Both in vivo [92] and in vitro [93] studies have confirmed an extensive BH of dietary EPA and DHA that was greater than 90%. In contrast to ALA, these PUFA are not completely hydrogenated into SFA, but numerous intermediates are produced including a majority of UFA and much lesser amounts of SFA [94]. The most recent in vitro study [95] suggests that while the reduction of the double bond at the closest position to the carboxyl group is the main BH pathway of EPA and DPA (Figure 4), this process is much less important for DHA. In addition, these authors stated that the possible interspecies differences between bovine and ovine BH of n-3 LC-PUFA is directly correlated with slower and less complete BH observed in cattle, especially for EPA and DPA. However, the specific pathways for BH of individual n-3 LC-PUFA still remain unclear.

Apart from ruminal BH, given the relatively low absorption rate from the small intestine into the mammary gland at 49% for ALA, and ranging from 14% to 33% for EPA, and from 13% to 25% for DHA [17], it is not surprising that the proportion of these PUFA in dairy products is generally very low. Principal strategies for increasing n-3 PUFA in milk and milk products, therefore, have been to minimize the biohydrogenation effects of ruminal microbes and/or improving the absorption rate of these FA into the mammary gland.

## 4. Recent Attempts to Increase n-3 PUFA Content In Dairy-Derived Products

Up to the present time, the nutritional manipulation of feeding regimes and supplementation with lipid sources containing high amounts of n-3 PUFA [17,96] are the major approaches to improving n-3 PUFA content in dairy products. In contrast, current efforts to employ genetic programmes in this theme have not yet yielded significant enhancement because the FA profile of milk processed products primarily depends on the FA composition of raw milk [97,98,99]. Therefore, current studies mostly focus on milk content as the principal route of increasing n-3 PUFA in other processed products.

### 4.1. Feeding Regime

Previous studies had demonstrated that feeding regime, particularly changes in forage sources and feeding systems, had significant effects on shorter chain n-3 PUFA, but minor effects on n-3 LC-PUFA concentrations in both dairy ewes and cows (Table 3). This is because lipids from pasture sources contain abundant amounts of ALA [100,101], but not EPA, DHA and DPA. For example, ALA content of fresh ryegrass varieties, a popular pasture used for ruminants worldwide, ranges from 62 to 74% of total fatty acids [102]. However, the pasture conservation processes, particularly grass wilting in the field, generally cause the oxidative loss of forage PUFA, subsequently and markedly reducing the content of ALA in hay or silage [100]. Wilting ryegrass 24 h in glasshouse, for instance, reduced the percentage of ALA by 33% compared to unwilted grass [103]. Therefore, dairy ruminants that are kept in grazing systems or have free access to fresh grass produced much higher proportions of ALA in milk compared with animals fed conserved grass (hay and silage) [104,105,106,107]. These results appear to be supported by the higher ALA intake of animals fed or grazed on fresh pastures.

The transfer of n-3 PUFA from forage into milk and milk products is also influenced by forage species (Table 3). Grazing dairy cows on diverse alpine pastures produced more ALA in their milk than on ryegrass-dominated paddocks (1.15 vs. 0.70 g/100 g FA) [105]. Both Addis et al. [109] and Bonanno et al. [110] reported the greatest concentration of ALA in sheep milk and cheese from ewes grazed on Sulla pasture, versus other common forages including ryegrass, burr medic and daisy forb. Guzatti et al. [111] showed higher levels of ALA in ewe milk for animals fed on clover silage compared with lucerne silage (0.92 vs. 0.70 g/100 g FA). Disparities observed between forage species in the transfer of n-3 PUFA into milk in these studies were not correlated with ALA intake, but were associated with variation in condensed tannin content in the forages. The most possible mechanisms and effects of the condensed tannins were explained by Cabiddu et al. [112], in which tannins inhibited rumen microbial activities, thus ultimately lowering the PUFA biohydrogenation process in the rumen. The attempt to reduce microbial species involved in biohydrogenation such as *B. proteoclasticus* has been implemented with limited success due to many factors. For more details, see comprehensive coverage by Lourenco et al. [113].

### 4.2. Lipid Supplementation

Lipid supplementation has been used as an effective tool to improve animal performance due to its significant energy contribution [101], and it can also alter milk fat composition because of the high content of essential FA [114,115]. Fish oils and marine products, oilseeds and vegetable oils are the main sources that have been employed in ruminant diets to enhance the concentrations of health beneficial n-3 PUFA and n-3 LC-PUFA in milk and milk products [101].

#### 4.2.1. Oil Seed and Vegetable Oil

Plant-derived fat is the most common fat source in ruminant supplements, and includes both oilseeds and extracted vegetable oils. This is because these materials not only contain a high concentration of PUFA [116], protein and energy [117], but are also more readily available and cheaper than other (marine) sources [22]. Therefore, a number of studies have examined the effects of oilseed and vegetable oils on the concentration of health beneficial n-3 PUFA in both bovine and ovine milk products (Table 4). Based on previously reported results, the addition of flaxseed or linseed supplements in ruminant diets is a more effective strategy to enrich milk n-3 PUFA compared to other plant fat supplementation methods (Table 4). Due to its very high content in ALA at approximately 53% of all FA [118], cows or sheep supplemented with flaxseed had substantial enhancement of this shorter chain n-3 PUFA in milk products (Table 4).

Oil infusion is also considered an effective form of providing plant oil supplements that increases the escape rate of UFA from the BH of rumen microbes, thus enhancing the availability of n-3 PUFA for absorption [17]. Khas et al. [124] reported that adding 160 g/day of infused free ALA in the diet for lactating cows increased ALA content in milk by 41-fold, and also resulted in significant increases in milk EPA and DPA by two-fold and three-fold, respectively. However, supplementation with vegetable seed and oils only marginally increased milk EPA, DHA, and DPA in both bovines and ovines, with the percentages of these FA often lower than 0.1 g/100g FA (Table 4). These findings indicated that the endogenous biosynthesis pathway of these n-3 LC-PUFA from dietary ALA in dairy animals is limited.

#### 4.2.2. Marine Lipid Sources

Feeding dairy animals with marine oil resulted in the highest n-3 LC-PUFA concentration in milk and milk products (Table 5) among all types of lipid supplements examined. Previous studies also confirmed the efficiency of utilising rumen-protected forms of marine products that were markedly higher than in the untreated controls; mainly as a result of the lesser extent of ruminal biohydrogenation with the rumen-protected diets [125]. Kitessa et al. reported that the content of EPA and DHA, which are generally scanty in milk (Table 3 and Table 4), could be increased by supplementing both dairy cattle [126] and ewes [127] with rumen-protected fish oil. The proportion of DHA, the most essential n-3 LC-PUFA, observed in these studies, exceeded 1% of the total FA. Similarly, an effective incorporation rate of DHA from a marine algae supplement, an alternative to fish oil into milk, was also confirmed by a number of studies (Table 5). This transfer rate appears to be higher as observed in ovine [128] than in bovine [129]. Results presented in Table 5 also indicate that supplementing fish oil is more advantageous than marine algae in terms of improving milk EPA and DPA content.

Recent focus on achieving quantitatively significant amounts of n-3 PUFA per standard serve of milk and milk products has occurred [45,46]. This absolute FA concentration data may be more accurate than the proportion (expressed as %FA) itself, since the fat percentage of milk from different species varies widely [130], and such quantitative data can potentially assist consumers in purchasing decisions. One serve of fresh milk produced from grazing ewes supplemented with rumen-protected EPA + DHA contains 62 mg of total n-3 LC-PUFA, three-fold higher than the control group [45]. This result is higher than the concentration of total EPA + DHA + DPA in one serve of cooked lamb meat (55 mg) reported by Flakemore et al. [131]. In achieving 60 mg/serving, this sheep milk can also be considered as achieving a “good source” level of n-3 LC-PUFA, adhering to Food Standards Australia and New Zealand (FSANZ) [132]. Although the inclusion of fish oil into ruminant diets might have a negative effect on meat quality such as possible rancidity and abnormal flavour in cooked or grilled lamb [133], no side effects on milk and milk products have been reported. Nguyen et al. [46] observed no differences in sensory eating traits between ripened cheese processed from milk produced by dairy sheep supplemented with rumen-protected marine source and the unsupplemented group. However, the higher cost of the marine oil source possibly limits its utilization as a routine supplementation for dairy ruminants [101].

### 4.3. Genetic Manipulation as a Potential Tool for the Enrichment of Dairy Products with n-3 PUFA

Attempts at understanding and estimating genetic parameters influencing milk FA content that may be beneficial for human health had been made a decade ago [146,147]. Up to the present time, low heritabilities (<0.1) for individual n-3 PUFA (Table 6) were consistently reported in dairy cows [147,148,149] and dairy sheep [150], indicating a low impact of genetics or breed on the concentration of n-3 PUFA. This observation probably arises because the fatty acids longer than 18 carbon chains are not de novo synthesized in the mammary gland, but are circulated from the blood which contains lipids that originated from the diet [94]. Moderate heritabilities for the whole group of n-3 PUFA were reported by Boichard et al. [151] and Maroteau et al. [152], and could be explained by major contribution of the shorter chain n-3 PUFA. First identified in the human genome in 2000 [153], fatty acid desaturase 1 and 2 (FADS1 and FADS2) are considered as the major candidate genes that regulate the endogenous synthesis of n-3 LC-PUFA from ALA in mammals including ruminants [154,155,156,157,158]. The first effort to define the association between these two encoding genes and n-3 PUFA in the milk of Holstein cows [159] found that three significant single nucleotide polymorphism (SNP) markers within FADS1 and FADS2 were associated with EPA. Apart from the two well-characterized FADS1 and FADS2 genes, Ibeagha-Awemu et al. [160] uncovered more potential candidate genes with several novel SNPs that were significantly associated with milk EPA and DPA. Consequently, by employing these potential genetic markers, future research can investigate the specific relationships between combining genetics and other environmental strategies such as nutritional supplementation for elevating n-3 LC PUFA in milk.

## 5. Conclusions

Since first being reported some four decades ago, the number of studies confirming the beneficial effects of n-3 PUFA on reducing the risk of chronic diseases including cardiovascular disease, cancer, mental illnesses, age-related cognitive decline, and inflammatory diseases were over and above those that found neutral or adverse effects. Specific mechanisms with respect to the independent and/or supplementary influences of n-3 PUFA on human health, however, remain controversial and need further investigations. Consumption of seafood as the major source of n-3 LC-PUFA is still generally insufficient, resulting in the need for enhancing the concentration of these beneficial n-3 LC-PUFA in other common dietary sources for human consumption. Attempts to increase the content of n-3 LC-PUFA in milk and milk products, which are important components of the human diet, has therefore been a major research focus for the last two decades. Current studies generally demonstrate that the processes of lipolysis combined with dietary lipid BH in the rumen are the main obstacles in modulating n-3 LC-PUFA content of dairy products. In addition, the biosynthetic pathway of these FA from the precursor ALA, is limited, with only minor conversion occurring. Thus, supplementing ruminants with marine-derived sources in a rumen-protected form is at this time, one of the best strategies to increase n-3 LC-PUFA content in milk and its processed products. Yet, there seems to be a lack of evaluation regarding the economic efficiency of different supplemented ingredients utilized in the current research. We propose that further studies are required to better elucidate the n-3 LC-PUFA biosynthesis pathway from ALA, together with the BH mechanism of individual n-3 LC-PUFA. A more comprehensive knowledge of these aspects would be a guiding principle for further research on increasing the escape rate of dietary PUFA from the rumen and their absorption rate from the small intestine into milk. Genetically modifying oilseeds as an alternative and sustainable source of dietary n-3 LC-PUFA for ruminants should be taken into account when future supplies of fish oil may not be sufficient. Finally, activities of regulated genes on the concentration of n-3 PUFA and their reaction to non-genetic factors such as lipid supplementation for specific dairy species also needs to be better defined. Identified knowledge gaps regarding the activities of candidate genes regulating the concentrations of n-3 PUFA and the responses of ruminants to specific lipid supplementation regimes are also critical to a greater understanding of nutrition-genetics interactions driving lipid metabolism.

## Figures and Tables

**Table 1:** Common food sources of ALA (18:3n-3, as gram per serving).

Item	Unit	ALA
Flaxseed oil	g/tbsp	7.26
Chia seed	g/ounce	5.06
English walnuts	g/ounce	2.57
Whole flaxseed	g/tbsp	2.35
Canola oil	g/tbsp	1.28
Soybean oil	g/tbsp	0.92
Black walnut	g/ounce	0.76

Data from Office of Dietary Supplements, National Institute of Health (NIH) [28]. Tbsp denotes tablespoon.

**Table 2:** Content of n-3 LC-PUFA in common seafood and other animal sources.

Item	Unit	EPA	DHA	DPA	Total n-3 LC-PUFA	Reference
**Wild seafood**						Nichols et al. [1]
Fish	mg/150 g	-	-	-	350	
Shellfish	mg/150 g	-	-	-	225	
Prawns	mg/150 g	-	-	-	180	
Lobster	mg/150 g	-	-	-	160	
**Farmed fish**						
Atlantic salmon	mg/100 g	-	-	-	980	Nichols et al. [38]
Barramundi	mg/100 g	-	-	-	790	
**Other animal sources**						
Beef	mg/100 g	15	12	20	47	Garcia et al. [39]
Chicken breast	mg/100 g	-	-	-	62.04	Konieczka et al. [40]
Pork	mg/100 g	23.3	3.9	21.1	48.3	Dugan et al. [41]
Feedlot lamb meat	mg/100 g	17.9	4.9	15.6	38.4	Nguyen et al. [42]
		28.9	13.3	19.6	61.8	Le et al. [43]
Grazing lamb meat	mg/100 g	25	7.1	23.7	55.8	Le et al. [44]
Sheep milk	mg/250 mL	17.8	19.8	24.1	61.7	Nguyen et al. [45]
Sheep cheese	mg/40 g	14.3	12.8	17.1	44.2	Nguyen et al. [46]
Cow milk	mg/100 g	3.3	-	4.4	-	Benbrook et al. [47]

**Table 3:** Effect of pasture feeding regimes on n-3 PUFA content of milk (g/100 g fatty acids).

Forage Source/Feeding System	Species	ALA	EPA	DHA	DPA	References
Ryegrass-dominated pastures	Bovine	0.703	0.083	0.009	0.109	Leiber et al. [105]
Freshly harvested ryegrass		0.619	0.073	0.009	0.113	
Alpine pastures		1.146	0.083	0.009	0.120	
Freshly harvested Alpine		0.950	0.083	0.010	0.118	
Silage-concentrate diet (control) ^1^		0.516	0.063	ND	0.082	
Ryegrass pasture	Bovine	0.68	0.05	0.02	0.07	Mohammed et al. [107]
Freshly harvested ryegrass		0.82	0.07	0.02	0.08	
Ryegrass silage		0.34	0.05	0.02	0.09	
Indoor hay based diet	Bovine	0.72	0.08	-	0.147	Coppa et al. [119]
Rotational grazing system		0.727	0.070	-	0.137	
Continuous grazing system		0.940	0.087	-	0.150	
Indoor conventional system	Bovine	0.579	0.072	-	0.118	Stergiadis et al. [120]
Indoor organic system		1.199	0.098	-	0.098	
Mixed forage ^2^	Bovine	0.47	-	-	-	Liu et al. [121]
Corn stalk1 diet (35%)		0.58	-	-	-	
Corn stalk2 diet (53.8%)		0.63	-	-	-	
Daisy forb − winter	Ovine	1.62	-	-	-	Addis et al. [109]
Ryegrass − winter		1.47	-	-	-	
Burr medic − winter		2.19	-	-	-	
Sulla − winter		2.98	-	-	-	
Daisy forb − spring		1.26	-	-	-	
Ryegrass − spring		1.44	-	-	-	
Burr medic − spring		1.84	-	-	-	
Sulla − spring		3.15	-	-	-	
Pasture	Ovine	1.07	0.06	-	0.13	Gomez-Cortes et al. [104]
Pasture + oat grain		0.59	0.05	-	0.12	
Total mixed ration ^3^		0.33	0.03	-	0.06	
Grass hay (in door)	Ovine	1.31	0.19	0.30	-	Mierlita [106]
Part-time grazing		2.06	0.28	0.39	-	
Pasture	Ovine	2.09	0.30	0.37	-	Mierlita et al. [122]
Pasture + standard concentrate ^3^		1.04	0.11	0.18	-	
Pasture	Ovine	0.44	0.01	0.07	0.13	Mohamed et al. [123]
Pasture + concentrate		0.24	0.00	0.12	0.07	
Concentrate		0.21	0.00	0.00	0.08	
Red clover silage	Ovine	0.92	0.05	-	0.09	Guzatti et al. [111]
Lucerne silage		0.70	0.05	-	0.09	

^1^ The control diet contained 60% ryegrass silage, 30% maize silage and 10% grass hay on dry matter basis. ^2^ Mixed forage contained 26.7% corn silage, 23.4% alfalfa hay and 3.7% Chinese wild rye on dry matter basis. ^3^ Total mixed ration contained concentrate and forage in proportion of 80:20.

**Table 4:** Effect of supplementing ruminants with plant-derived dietary sources on n-3 PUFA concentration in milk and milk products (g/100 g fatty acids).

Diet	Species	Product	ALA	EPA	DHA	DPA	References
Control	Bovine	Milk	0.61	0.09	-	0.07	Khas et al. [124]
40 g/day infused LNA-rich fatty acid			6.49	0.18	-	0.12	
80 g/day infused LNA-rich fatty acid			12.42	0.22	-	0.16	
120 g/day infused LNA-rich fatty acid			18.75	0.21	-	0.29	
160 g/day infused LNA-rich fatty acid			25.38	0.22	-	0.23	
Control	Bovine	Milk	0.75	0.003	0.001	-	Caroprese et al. [134]
Whole flaxseed			0.81	0.022	0.001	-	
Control	Bovine	Milk	0.41	0.05	-	0.05	Dai et al. [135]
Rapeseed oil			0.38	0.06	-	0.04	
Peanut oil			0.33	0.06	-	0.06	
Sunflower seed oil			0.32	0.06	-	0.05	
Control	Bovine	Milk	0.83	0.09	0.01	0.13	Otto et al. [136]
25mL/kg DM ^1^ Canola oil			0.85	0.09	0.01	0.14	
35 mL/kg DM canola oil			0.95	0.08	0.01	0.12	
50 mL/kg DM canola oil			0.97	0.08	0.00	0.11	
Control	Bovine	Milk	0.28	0.02	-	-	Cattani et al. [137]
500 g/day extruded flaxseed			0.50	0.02	-	-	
1000 g/day extruded flaxseed			0.59	0.02	-	-	
Linseed oil	Bovine	Milk	0.249	0.019	-	0.014	Li et al. [138]
Safflower oil			0.180	0.013	-	0.007	
Control	Bovine	Milk	0.19	0.012	0.004	0.037	Welter et al. [139]
3% Canola oil			0.36	0.011	0.003	0.034	
6% Canola oil			0.35	0.011	0.003	0.033	
Control	Bovine	Milk	0.19	-	0.019	-	Vanbergue et al. [140]
Extruded linseed			0.51	-	0.008	-	
Palm oil	Ovine	Milk	0.52	0.04	0.02	0.08	Bodas et al. [141]
Olive oil			0.36	0.03	0.02	0.06	
Soybean oil			0.53	0.03	0.02	0.07	
Linseed oil			1.07	0.05	0.04	0.11	
Control	Ovine	Milk	1.21	0.05	0.05	-	Mughetti et al. [142]
100 g extruded linseed			1.65	0.06	0.09	-	
200 g extruded linseed			2.26	0.06	0.10	-	
Control	Ovine	Milk	0.57	0.07	0.05	0.08	Caroprese et al. [143]
Seaweed			0.59	0.06	0.04	0.08	
Whole flaxseed			1.53	0.08	0.05	0.09	
Seaweed + Whole flaxseed			1.32	0.08	0.06	0.10	
Control	Ovine	Milk	0.62	0.08	0.04	0.08	Nguyen et al. [45]
Canola oil			0.73	0.09	0.06	0.13	
Rice bran oil			0.51	0.07	0.04	0.10	
Flaxseed oil			1.74	0.11	0.06	0.15	
Safflower oil			0.67	0.07	0.06	0.10	
Control	Ovine	Milk	0.31	0.04	0.02	0.08	Parentet et al. [144]
Canola oil			0.26	0.03	0.02	0.07	
Sunflower oil			0.24	0.03	0.02	0.07	
Castor oil			0.28	0.05	0.01	0.08	
Control	Bovine	Cheese	0.29	0.02	-	-	Cattani et al. [137]
500 g/day extruded Flaxseed at			0.50	0.02	-	-	
1000 g/day extruded Flaxseed at			0.61	0.02	-	-	
Palm oil	Ovine	Cheese	0.54	0.04	0.02	0.07	Bodas et al. [141]
Olive oil			0.36	0.03	0.03	0.06	
Soybean oil			0.51	0.03	0.02	0.06	
Linseed oil			1.04	0.03	0.03	0.09	
Control	Ovine	Cheese	1.18	0.02	0.03	-	Mughettiet al. [142]
100 g extruded linseed			1.84	0.04	0.05	-	
200 g extruded linseed			2.02	0.04	0.06	-	
Control	Ovine	Cheese	0.71	0.11	0.06	0.12	Nguyen et al. [46]
Canola oil			0.79	0.11	0.06	0.13	
Rice bran oil			0.63	0.10	0.06	0.12	
Flaxseed oil			1.30	0.11	0.06	0.13	
Safflower oil			0.71	0.11	0.08	0.13	
Control	Ovine	Yogurt	0.0	-	-	-	Bianchi et al. [145]
2% Palm oil			0.0	-	-	-	
4% Palm oil			0.28	-	-	-	
6% Palm oil			0.31	-	-	-	

^1^ DM: dry matter.

**Table 5:** Effect of supplementing ruminants with dietary marine sources on n-3 PUFA concentration of milk and milk products (g/100 g fatty acids).

Diet	Species	Product	ALA	EPA	DHA	DPA	References
Control	Bovine	Milk	0.54	-	0.00	-	Franklin et al. [161]
Protected algae			0.49	-	0.76	-	
Unprotected algae			0.47	-	0.46	-	
Control	Bovine	Milk	0.86	0.0	0.0	-	Kitessa et al. [126]
Rumen-protected tuna oil			1.28	0.61	1.09	-	
Control	Bovine	Milk	0.21	0.03	0.00	0.07	Shingfield et al. [162]
Fish oil and sunflower oil			0.23	0.11	0.07	0.16	
Control	Bovine	Milk	0.50	-	0.09	-	Boeckaert et al. [129]
Marine algae			0.42	-	1.01	-	
ABO/ABO ^1^	Bovine	Milk	14.4	0.22	-	0.22	Kazama et al. [163]
RUM/ABO ^2^			4.78	0.14	-	0.22	
RUM/RUM ^3^			2.33	0.09	-	0.12	
ABO/RUM ^4^			11.6	0.16	-	0.18	
Control	Bovine	Milk	0.75	0.003	0.001	-	Caroprese et al. [134]
Fish oil			0.84	0.060	0.117	-	
Control	Bovine	Milk	0.45	0.06	0.10	-	Vargas-Bello-Pérez et al. [164]
Fish oil			0.62	0.10	0.21	-	
Fish oil + palm oil			0.69	0.09	0.14	-	
Control	Bovine	Milk	0.41	0.06	0.03	0.09	Kairenius et al. [165]
Ultrarefined fish oil at 75 g/day			0.38	0.06	0.03	0.08	
Ultrarefined fish oil at 150 g/day			0.39	0.07	0.05	0.10	
Ultrarefined fish oil at 300 g/day			0.48	0.17	0.10	0.18	
Control	Bovine	Milk	0.19	-	0.019	-	Vanbergue et al. [140]
Microalgae DHA Gold^®^			0.25	-	0.444	-	
Extruded linseed + DHA Gold^®^			0.46	-	0.170	-	
Control	Ovine	Milk	0.33	ND	ND	ND	Papadopoulos et al. [128]
Low algae (23.5 g)			0.31	0.04	0.43	0.21	
Medium algae (47 g)			0.33	0.12	0.69	0.28	
High algae (94 g)			0.25	0.21	1.24	0.31	
Control	Ovine	Milk	0.53	0.05	0.03	0.10	Toral et al. [166]
Sunflower oil (SO)			0.41	0.04	0.02	0.07	
SO + 8 g/kg DM of Marine Algae			0.37	0.05	0.17	0.10	
SO + 16 g/kg DM of Marine Algae			0.36	0.09	0.46	0.13	
SO + 24 g/kg DM of Marine Algae			0.34	0.10	0.57	0.15	
Sunflower oil	Ovine	Milk	0.49	0.04	0.05	0.10	Bichi et al. [167]
Sunflower oil + Marine algae			0.48	0.06	0.38	0.12	
Control	Ovine	Milk	0.62	0.08	0.04	0.08	Nguyen et al. [45]
Rumen-protected EPA + DHA oil			0.74	0.17	0.19	0.23	
Control	Bovine	Cheese	0.01	0.05	0.09	-	Vargas-Bello-Pérez et al. [164]
Fish oil			0.02	0.12	0.34	-	
Fish oil + palm oil			0.01	0.09	0.18	-	
Control	Ovine	Cheese	0.71	0.11	0.06	0.12	Nguyen et al. [46]
Rumen-protected EPA + DHA			1.02	0.16	0.15	0.19	

^1^ ABO/ABO diet contains abomasal flax oil and hulls infusion. ^2^ RUM/ABO diet contains flax oil placed in the rumen and hulls infused in the abomasum. ^3^ RUM/RUM diet contains flax oil and hulls placed in the rumen and abomasal infusion of water. ^4^ ABO/RUM diet contains flax hulls administered in the rumen and abomasal flax oil infusion.

**Table 6:** Heritability estimates of major individual and group of n-3 fatty acids.

Breed	Species	Unit	ALA	EPA	DPA	n-3	Reference
Holstein-Friesians	Bovine	%FA	0.09	-	-	-	Stoop et al. [147]
Holsteins	Bovine	% FA	0.06	0.04	0.01	-	Bilal et al. [148]
Brown Swiss	Bovine	% FA	0.093	0.045	0.039	0.085	Pegolo et al. [149]
Sarda	Ovine	% FA	0.02	-	-	-	Correddu et al. [150]
Holstein	Bovine	% fat	-	-	-	0.26	Boichard et al. [151]
Saanen	Caprine		-	-	-	0.23	
Lacaune	Ovine		-	-	-	0.18	
Alpine	Caprine	% fat	-	-	-	0.28	Maroteau et al. [152]
Saanen	Caprine		-	-	-	0.25	
